# MAB21L1 promotes survival of lens epithelial cells through control of αB-crystallin and ATR/CHK1/p53 pathway

**DOI:** 10.18632/aging.204203

**Published:** 2022-08-10

**Authors:** Yuan Xiao, Jia-Wen Xiang, Qian Gao, Yue-Yue Bai, Zhao-Xia Huang, Xiao-Hui Hu, Ling Wang, David Wan-Cheng Li

**Affiliations:** 1 College of Life Sciences, Hunan Normal University, Changsha 410080, Hunan, China; 2 The State Key Laboratory of Ophthalmology, Zhongshan Ophthalmic Center, Sun Yat-Sen University, Tianhe, Guangzhou 510230, Guangdong, China; 3 Department of Basic Medicine, Guizhou University of Traditional Chinese Medicine, Guiyang 121212, Guizhou, China; 4 The Academician Work Station, Changsha Medical University, Changsha 410219, Hunan, China

**Keywords:** apoptosis, αB-crystallin, lens, cataract

## Abstract

The male abnormal gene family 21 (*mab21*), was initially identified in *C. elegans*. Since its identification, studies from different groups have shown that it regulates development of ocular tissues, brain, heart and liver. However, its functional mechanism remains largely unknown. Here, we demonstrate that Mab21L1 promotes survival of lens epithelial cells. Mechanistically, Mab21L1 upregulates expression of αB-crystallin. Moreover, our results show that αB-crystallin prevents stress-induced phosphorylation of p53 at S-20 and S-37 through abrogating the activation of the upstream kinases, ATR and CHK1. As a result of suppressing p53 activity by αB-crystallin, Mab21L1 downregulates expression of Bak but upregulates Mcl-1 during stress insult. Taken together, our results demonstrate that Mab21L1 promotes survival of lens epithelial cells through upregulation of αB-crystallin to suppress ATR/CHK1/p53 pathway.

## INTRODUCTION

The male abnormal gene family 21 (*mab21*), was first identified in *C. elegans* [1]. Since its identification, *mab21L1* gene was found to control development of both eye and brain [2–9]. Moreover, it also has a role in regulating axis and dorsal-ventral patterning as well as heart and liver development [3, 4, 10]. Mutations of the human *mab21L1* gene cause numerous ocular diseases including ocular coloboma, microcornea and cataract, neural defects, skeletal dysplasia and intellectual disability [7, 9, 11]. However, the functional mechanism of the *mab21L1* gene remains largely unknown [8, 12].

Apoptosis is one of the major causes for ocular diseases [13–16]. In lens system, we initially demonstrated that induced lens epithelial cell apoptosis appears to be a common cellular mechanism mediating stress-induced, non-congenital cataractogenesis [17–19]. Subsequently, a number of laboratories have confirmed that lens epithelial cell apoptosis is indeed actively involved in lens pathogenesis as demonstrated from *in vivo* animal model studies [20–25]. Moreover, transgenic studies with overexpression of various exogenous genes or disruption of several endogenous genes caused apoptosis followed by cataractogenesis or small eye during lens development [26–33].

In lens epithelial cells, apoptosis is mainly mediated by p53 and its downstream targets [26, 30, 33–49]. Bcl-2 family proteins play a crucial role in mediating endogenous apoptotic pathway [50–54], and among which Bak is an important pro-apoptotic protein [55–59] and Mcl-1 is a major anti-apoptotic regulator [60–63].

αB-crystallin is initially known as major lens structural protein that plays an essential role in maintaining the transparency of the ocular lens [64]. Later, αB-crystallin is found to be a member of the small heat shock protein family [65] and act as a molecular chaperone [66–73] and also displays autokinase activity [74, 75]. As a strong antiapoptotic regulator, αB-crystallin is initially shown to protect cells from osmotic [76], thermal [77] and oxidative insult [78]. Later, αB-crystallin is found to prevent induced apoptosis by various factors including staurosporine [54, 79–81], TNF [79, 81], UVA irradiation [81, 82], okadaic acid [38] and hydrogen peroxide [51]. Regarding the antiapoptotic mechanism, previous studies from our laboratory and others have demonstrated that αB-crystallin can directly interact with multiple targets including members of the caspase and Bcl-2 families as well as the tumor suppressor p53 to suppress apoptosis [38, 54, 82–89].

In the present study, we present evidence to show that the *mab21L1* gene has a role in promoting survival of lens epithelial cells. Mechanistically, overexpression of Mab21L1 upregulates expression of αB-crystallin. Moreover, we demonstrate that αB-crystallin prevents p53 phosphorylation at multiple sites through abrogating activation of the ATR and CHK1 kinases. As a result of suppressing p53 activity by αB-crystallin, Mab21L1 downregulates expression of Bak but upregulates Mcl-1 during okadaic acid treatment. It is well established that p53 plays an important role in regulating aging [90–92]. By regulating p53 activity, Mab21L1 may be involved in control of aging. Taken together, our results demonstrate that Mab21L1 promotes survival of lens epithelial cells through upregulation of αB-crystallin to suppress ATR/CHK1/p53 pathway.

## RESULTS

### Establishment of the stable lens epithelial cells expressing vector and Mab21L1

**Figure 1:**
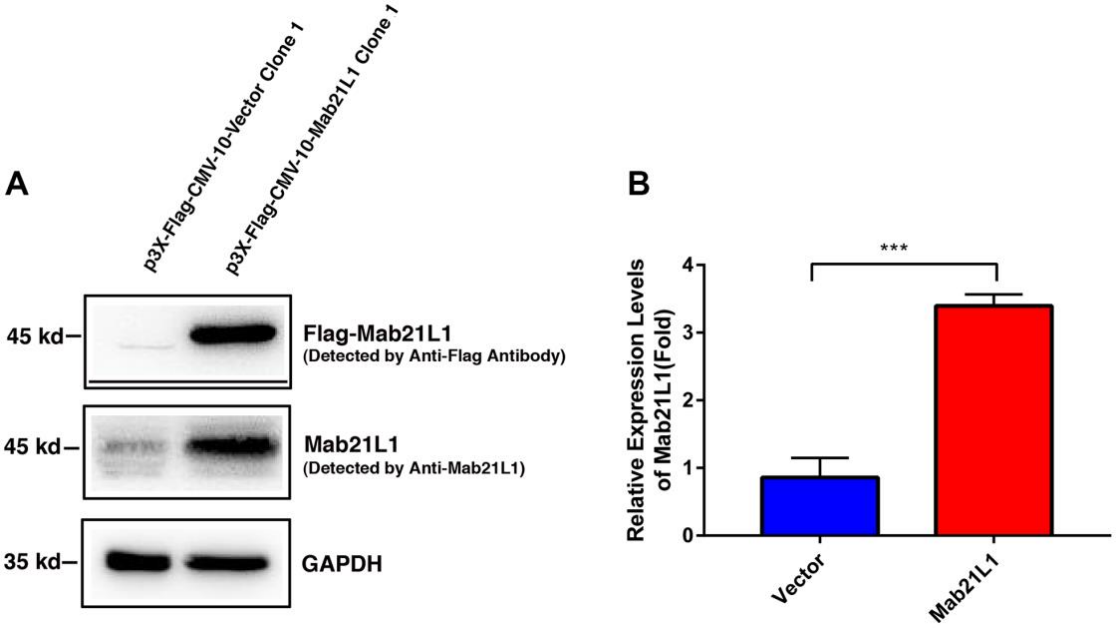
**Establishment of vector and MAB21L1 overexpression clones with mouse lens epithelial cells, αTN4-1 line.** The plasmids of P3X-Flag-CMV-10-Vector and P3X-Flag-CMV-10-MAB21L1 were transfected into αTN4-1 cells, respectively. After transfection, the P3X-Flag-CMV-10-Vector-αTN4-1 (Vector-αTN4-1 in short) cells and P3X-Flag-CMV-10-MAB21L1-αTN4-1 (MAB21L1-αTN4-1 in short) cells were screened with 600 μg/ml G418 for 4 weeks, and individual clones were obtained. Clone 1 of vector-αTN4-1 and MAB21L1-αTN4-1 cells were confirmed by Western blot analysis (**A**). (**B**) Quantitative results of the MAB21L1 protein expression levels in A by Image J software. N=3. *** *p*<0.001.

To explore the function of Mab21L1, we have overexpressed the human Mab21L1 cDNA in mouse lens epithelial cells αTN4-1 using the vector p3X-FLAG-CMV-10-Mab21L1 [with cDNA inserted in EcoRI (5’) and Kpn I (3’)] (Supplementary Figure 1). As shown in Figure 1A, 1B, αTN4-1 cells have very low level of endogenous Mab21L1, and overexpression of human Mab21L1 lead to over 2-fold upregulation of Mab21L1. Thus, we established two stable lines: vector-transfected cells, p3X-FLAG-CMV-10-αTN4-1 (Vector-αTN4-1 in short), and Mab21L1-transfected cells, p3X-FLAG-CMV-10-Mab21L1-αTN4-1 (Mab21L1-αTN4-1 in short). The clone 1 from both vector- and Mab21L1-transfected cells were used in most related experimental studies described below.

### Mab21L1 promotes survival of lens epithelial cells treated with okadaic acid

**Figure 2:**
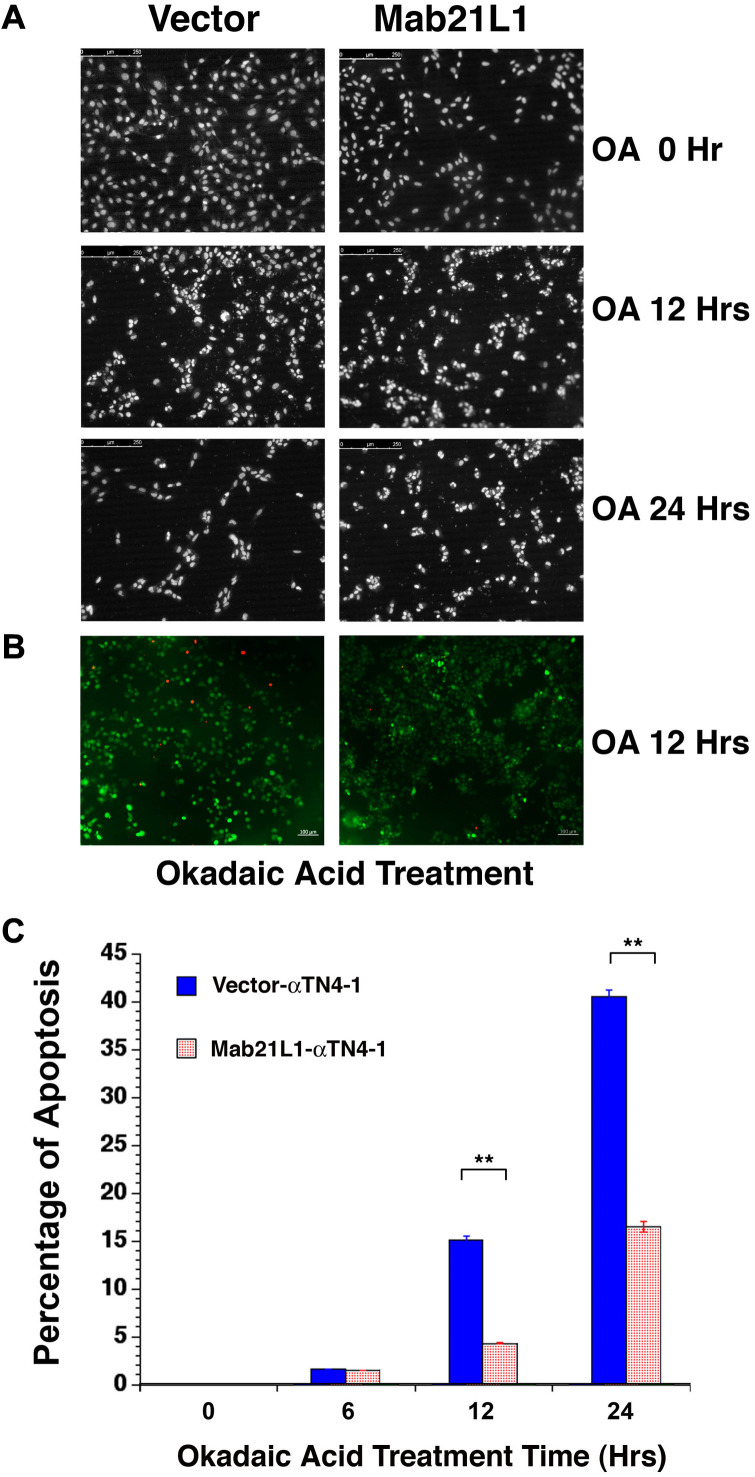
**Analysis of okadaic acid (OA)-induced apoptosis of vector-αTN4-1 (vector) and MAB21L1-αTN4-1 (Mab21L1) cells.** (**A**) Hoechst staining of OA-treated vector-αTN4-1 and MAB21L1-αTN4-1 cells for 0 to 24 hours. The apoptotic cells displayed fragmented or condensed nuclei, or were detached from the culture dishes, so that empty space appeared. Both vector-αTN4-1 and MAB21L1-αTN4-1 cells were grown to the density as shown in row one of Figure 2A (approximately 90% confluence, 0 Hr treatment), then subjected to 100 nM okadaic acid (OA) treatment with for 12 and 24 hrs. After OA treatment, the cells were processed for Hoechst staining as described (Mao et al., 2001). (**B**) Image of live cells (green color) and apoptotic cells (read color) of the vector-αTN4-1 and MAB21L1-αTN4-1 cells after 12 Hrs treatment by OA. (**C**) Quantitative results of apoptosis rate using live/dead assay as described (Wang et al., 2021). OA treatment for 12 hrs and 24 hrs induced about 15% and 41% apoptosis in vector-transfected cells, respectively. In contrast, only about 5% and 16% apoptosis were detected in MAB21L1-transfected cells after 12h and 24h-treatment with 100 nM OA. Note that MAB21L1 displayed the anti-apoptotic ability in αTN4-1 cells. Scale bar, 250 μm. N=3. ** *p*<0.01.

Next, we tested if Mab21L1 expression cells display any property in resisting stresses upon exposure to phosphatase inhibitor, okadaic acid [38]. Both vector- and Mab21L1-transfected cells were treated with 100 nM okadaic acid for 0 to 24 hours. As shown in Figure 2, cells expressing Mab21L1 were much resistant against 100 nM okadaic acid than vector-transfected cells. After 24-hour treatment by 100 nM OA, over 40% vector-transfected cells underwent apoptosis. In contrast, less than 20% cells were induced to undergo apoptosis (Figure 2A, 2C). The apoptotic nature was confirmed with live/dead assay in which the live cells were stained in green color and the dead cells were stained in red (Figure 2B). Thus, Mab21L1 can promote survival of the transfected lens epithelial cells. Mab21L1 also displayed clear protection against UVA irradiation-induced apoptosis (data not shown).

### Mab21L1 upregulates αB-crystallin during OA-induced apoptosis

**Figure 3:**
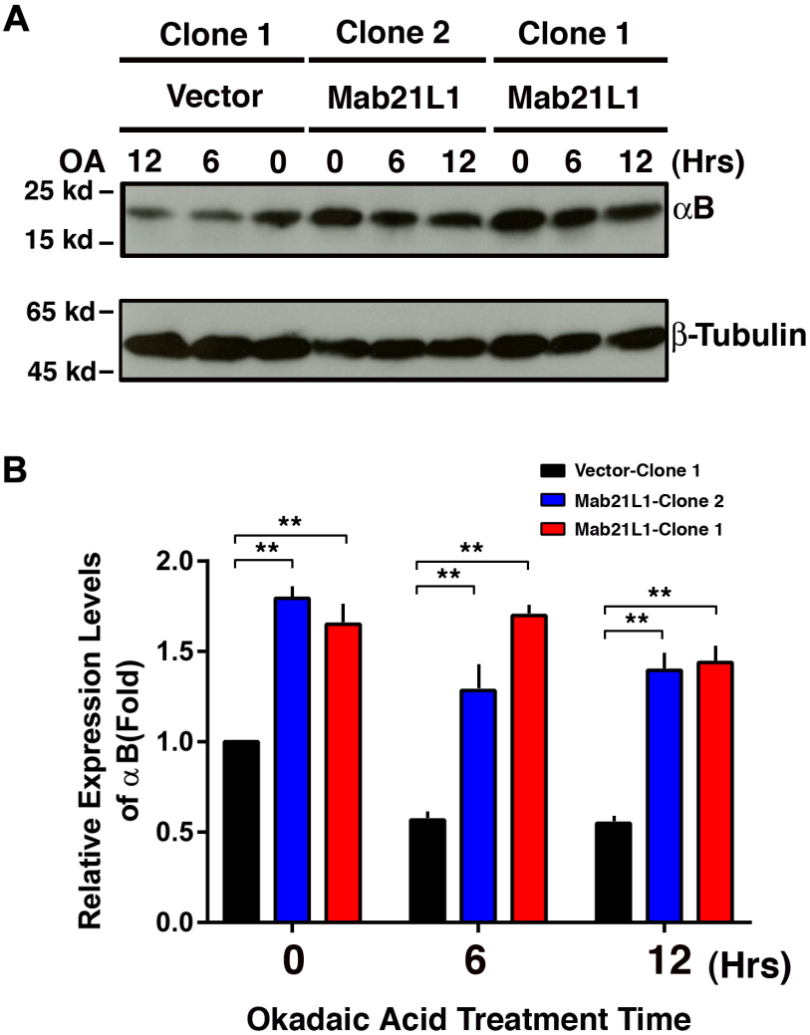
**Upregulation of αB-crystallin in Mab21L1-αTN4-1 cells and its attenuated degradation during okadaic acid (OA)-induced apoptosis of the Mab21L1-αTN4-1 cells.** Both vector-αTN4-1 and MAB21L1-αTN4-1 cells were grown to about 90% confluence and then subjected to 100 nM OA treatment for 12 and 24 hrs. Thereafter, the cells were harvested for extraction of total proteins which were used for analysis of αB-crystallin expression by Western blot analysis (**A**). Quantitative results of the αB-crystallin protein expression levels were analyzed by Image J software (**B**). Note that in the MAB21L1-αTN4-1 cell clones, the αB-crystallin protein expression level was much higher than that in the vector-αTN4-1 cell clone in the absence of 100 nM OA treatment. During OA treatment for 12 and 24 hrs, the degradation of αB-crystallin protein was much slower in MAB21L1-αTN4-1 cell clones than that in vector-αTN4-1 clone. N=3. ** *p*<0.01.

To explore how Mab21L1 could promote survival of lens epithelial cells against stress conditions including the okadaic acid and UVA irradiation, we examined expression of αB-crystallin during treatment by okadaic acid. As shown in Figure 3, cells expressing Mab21L1 displayed enhanced expression of αB-crystallin. Moreover, during okadaic acid treatment, in Mab21L1 expression cells, OA-induced downregulation of αB-crystallin was much slower than that in vector-transfected cells. Thus, Mab21L1 expression upregulates αB-crystallin, a small heat shock protein having strong antiapoptotic ability [38, 51, 54, 82–89].

### αB-crystallin prevents UVA-induced phosphorylation of p53 at S-20 and S-37

**Figure 4:**
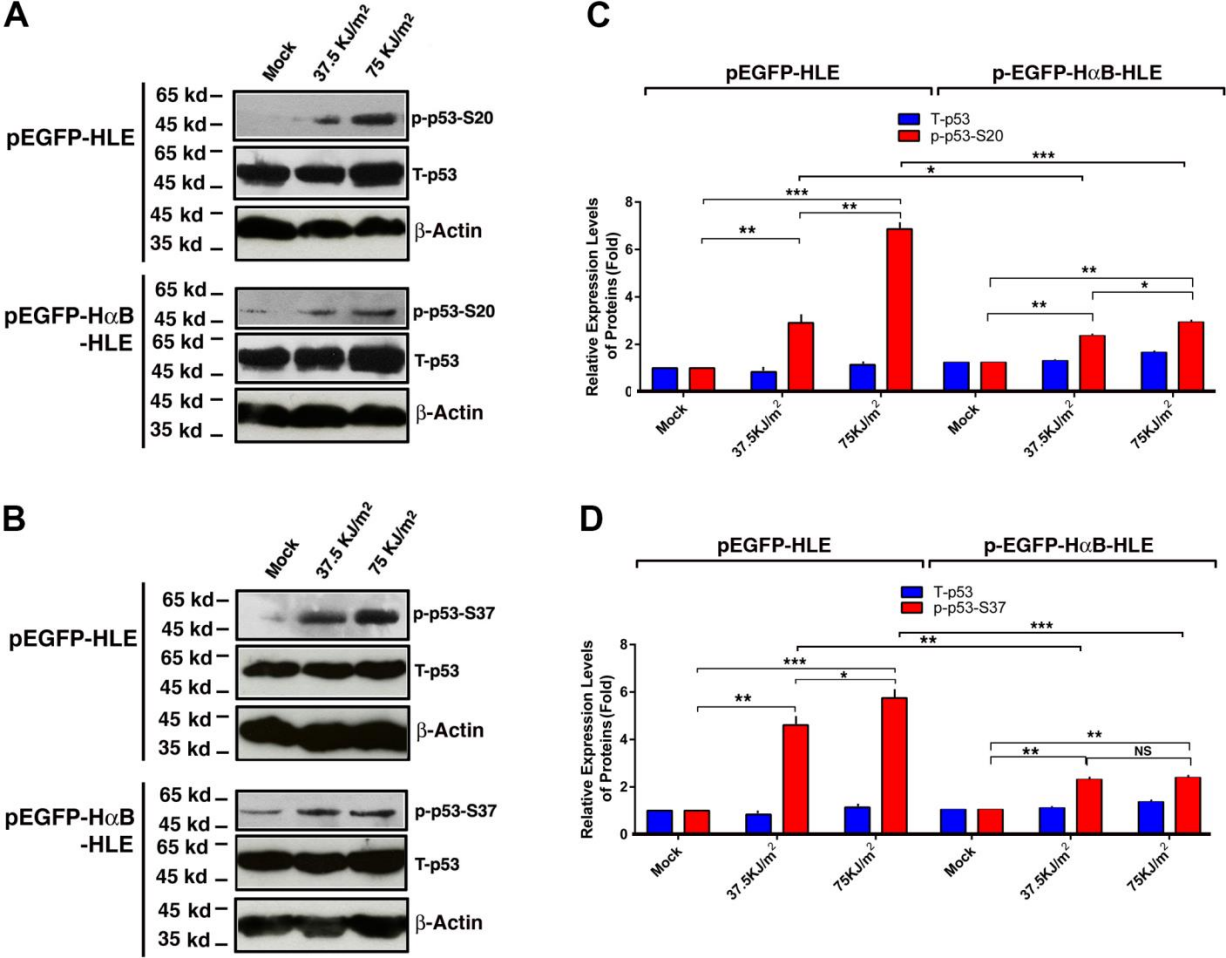
**αB-crystallin inhibited p53 hyperphosphorylation under UVA irradiation.** Both pEGFP-HLE and pEGFP-HαB-HLE cells (Mao et al., 2004) were grown to 90% confluence, then subjected to mock irradiation, 37.5 KJ/m2 and 75 KJ/m2 UVA irradiation, respectively. The irradiated cells were harvested for extraction of total proteins which were used for analysis of total p53 (T-p53) (**A**, **B**), phosphorylated p53 at S-20 (p-p53-S20) (**A**) and phosphorylated p53 at S-37 (p-p53-S37) (**B**) by Western blot analysis. Quantitative results of the T-p53, p-p53-S20 (**C**) and p-p53-S37 (**D**) levels were analyzed by Image J software. Note that UVA-induced much stronger p53 activity (phosphorylation at S-20 and S-37) in pEGFP-HLE cells than that in pEGFP-HαB-HLE cells. N=3. NS, not significant; *p<0.05, ** *p*<0.01, *** *p*<0.001.

We and others have previously shown that αB-crystallin can prevent apoptosis through regulation of multiple targets [38, 51, 54, 82–86]. To further explore the possible mechanisms how αB-crystallin prevents apoptosis, we examined the effects of αB-crystallin on p53 phosphorylation at S-20 and S-37. As shown in Figure 4, in vector-transfected cells (pEGFP-HLE), UVA irradiation induces significant hyperphosphorylation of p53 at S-20 and S-37. However, in αB-crystallin expression cells (pEGFP-HαB-HLE), UVA irradiation-induced hyperphosphorylation of p53 was much reduced. Thus, αB-crystallin can prevent UVA-induced hyperphosphorylation of p53 at S-20 and S-37.

### αB-crystallin prevents UVA-induced activation of check kinase 1 (CHK1)

**Figure 5:**
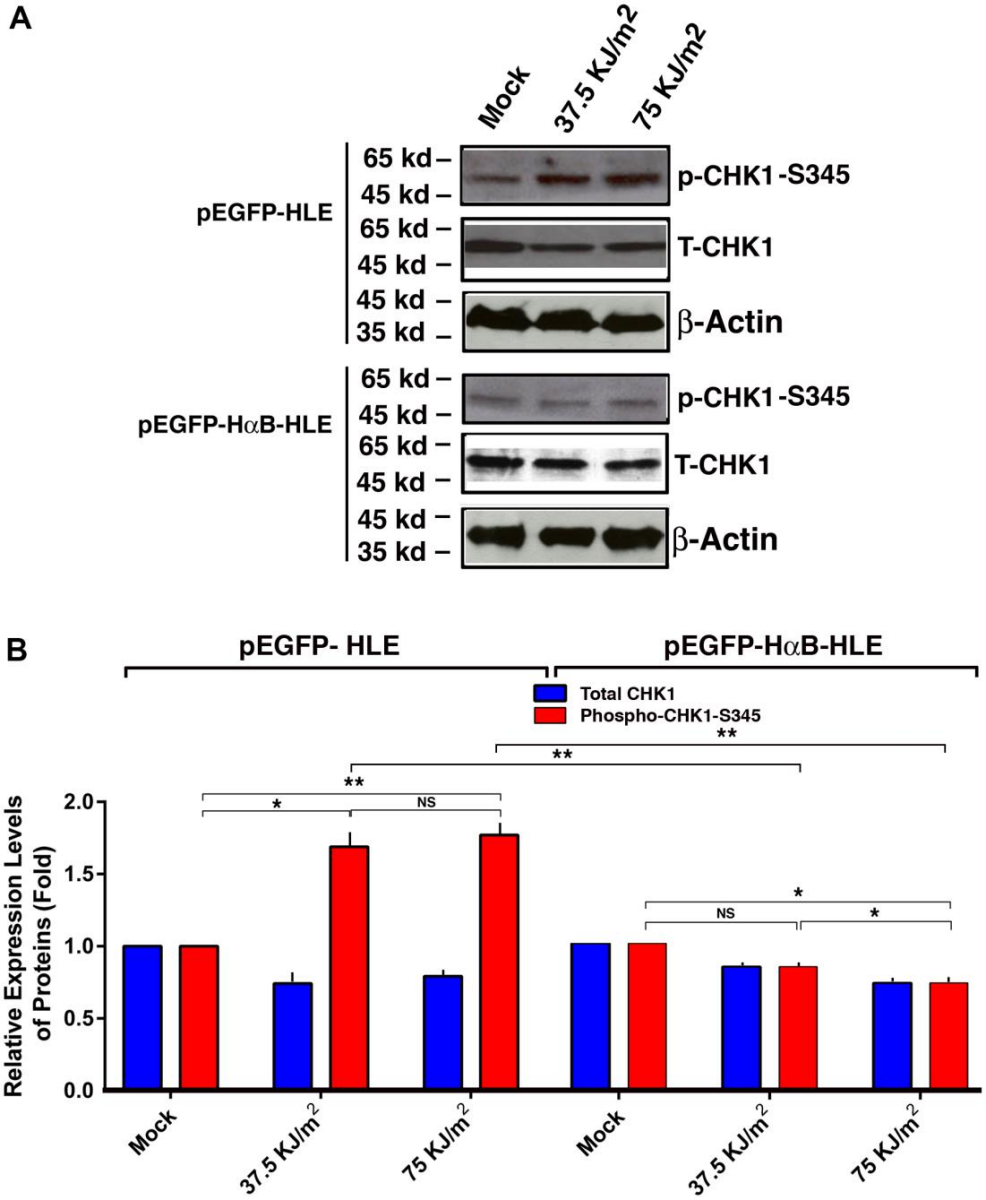
**αB-crystallin inhibited CHK1 activation under the UVA irradiation.** Both pEGFP-HLE and pEGFP-HαB-HLE cells (Mao et al., 2004) were grown to 90% confluence, then subjected to mock irradiation, 37.5 KJ/m2 and 75 KJ/m2 UVA irradiation, respectively. The irradiated cells were harvested for extraction of total proteins which were used for analysis of total CHK1 (T-CHK1, 54 kd), phosphorylated CHK1 at S-345 and β-Actin (loading control) levels (**A**) by Western blot analysis. Quantitative results of the T-CHK1 and p-CHK1-S345 against β-actin (loading control) levels in pEGFP-HLE cells and pEGFP-HαB-HLE cells (**B**) were analyzed by Image J software. Note that UVA induced significant upregulation of CHK1 activity (as reflected by phosphorylation at S345) in pEGFP-HLE cells. In contrast, in pEGFP-HαB-HLE cells, both total CHK1 and phosphorylated CHK1 at S345 were downregulated under UVA irradiation. N=3. NS, not significant. *p<0.05, ** *p*<0.01.

To explore how αB-crystallin prevents UVA-induced hyperphosphorylation of p53 at S-20 and S-37, we examined if αB-crystallin has any effect on the upstream kinase, check kinase 1 (CHK1). Previous studies have shown that CHK1 can phosphorylate p53 at these sites [93, 94]. As shown in Figure 5, UVA irradiation induced activation of CHK1 (as reflected by the enhanced phosphorylation at S-345) in vector-transfected cells. In αB-crystallin expression cells, however, activation of CHK1 was largely abrogated. Thus, αB-crystallin is capable of suppressing UVA irradiation-induced activation of CHK1.

### αB-crystallin prevents UVA-induced activation of ATR kinase

**Figure 6:**
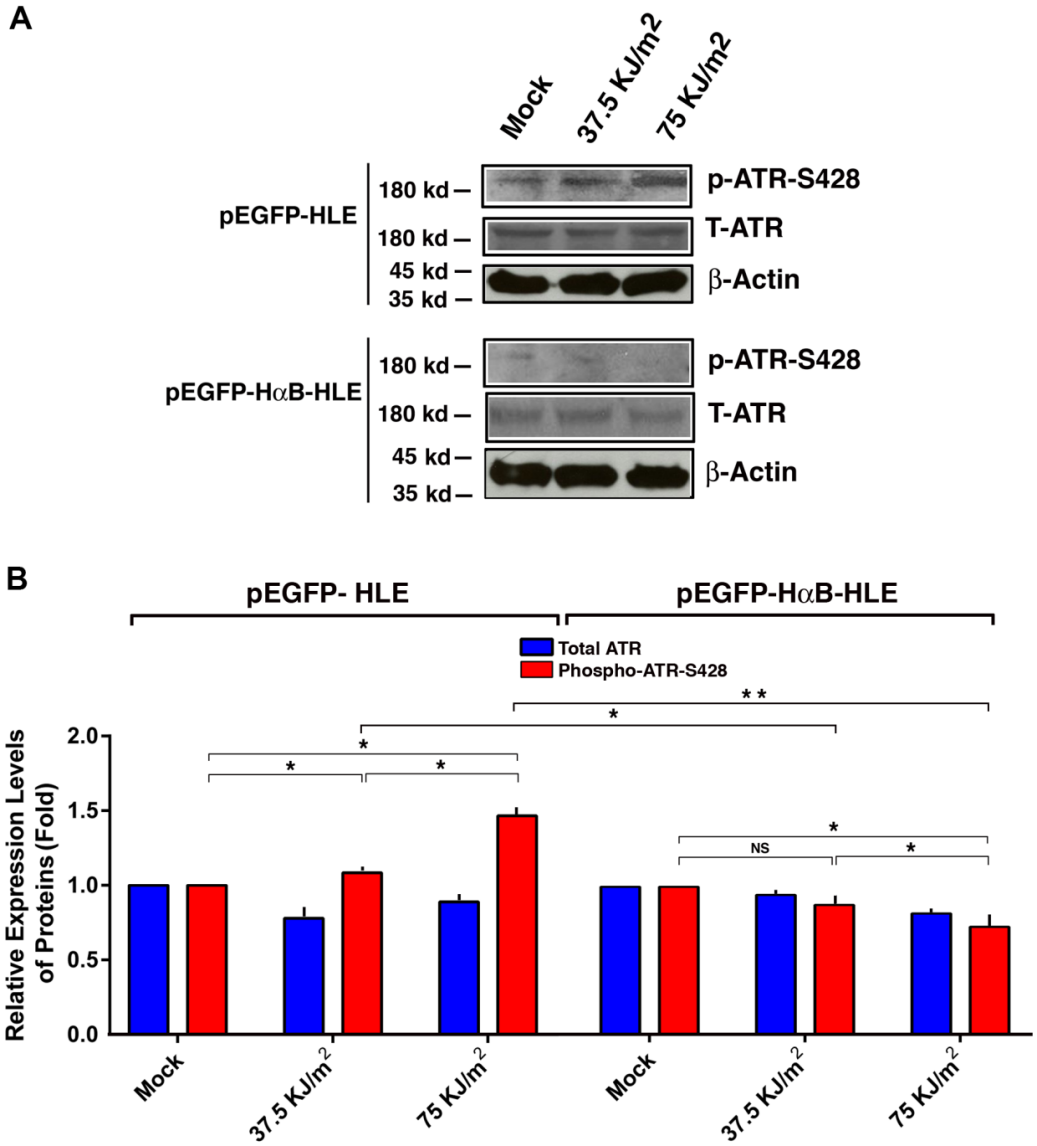
**αB-crystallin inhibited ATR activation under the UVA irradiation.** Both pEGFP-HLE and pEGFP-HαB-HLE cells (Mao et al., 2004) were grown to 90% confluence, then subjected to mock irradiation, 37.5 KJ/m2 and 75 KJ/m2 UVA irradiation, respectively. The irradiated cells were harvested for extraction of total proteins which were used for analysis of total ATR (T-ATR, 300 kd), phosphorylated ATR at S-428 and β-Actin (loading control) by Western blot analysis (**A**). Quantitative results of the T-ATR, and p-ATR-S428 levels against loading control in pEGFP-HLE cells and pEGFP-HαB-HLE cells (**B**) were analyzed by Image J software. Note that UVA induced significant upregulation of ATR activity (as reflected by phosphorylation at S428) in pEGFP-HLE cells. In contrast, in pEGFP-HαB-HLE cells, both total ATR and phosphorylated ATR at S428 were downregulated under UVA irradiation. N=3. NS, not significant. *p<0.05.

Since CHK1 is activated by the upstream kinase, ATR [95–98], we next examined if αB-crystallin has any effect on the upstream ATR kinase. As shown in Figure 6, UVA irradiation induced activation of ATR kinase (also reflected by the enhanced phosphorylation at S-428 in vector-transfected cells). In αB-crystallin expression cells, however, activation of ATR was totally abrogated. Thus, αB-crystallin is also capable of suppressing UVA irradiation-induced activation of ATR kinase.

### Mab21L1 downregulates Bak during OA-induced apoptosis and silence of Bak attenuates OA-induced apoptosis

**Figure 7:**
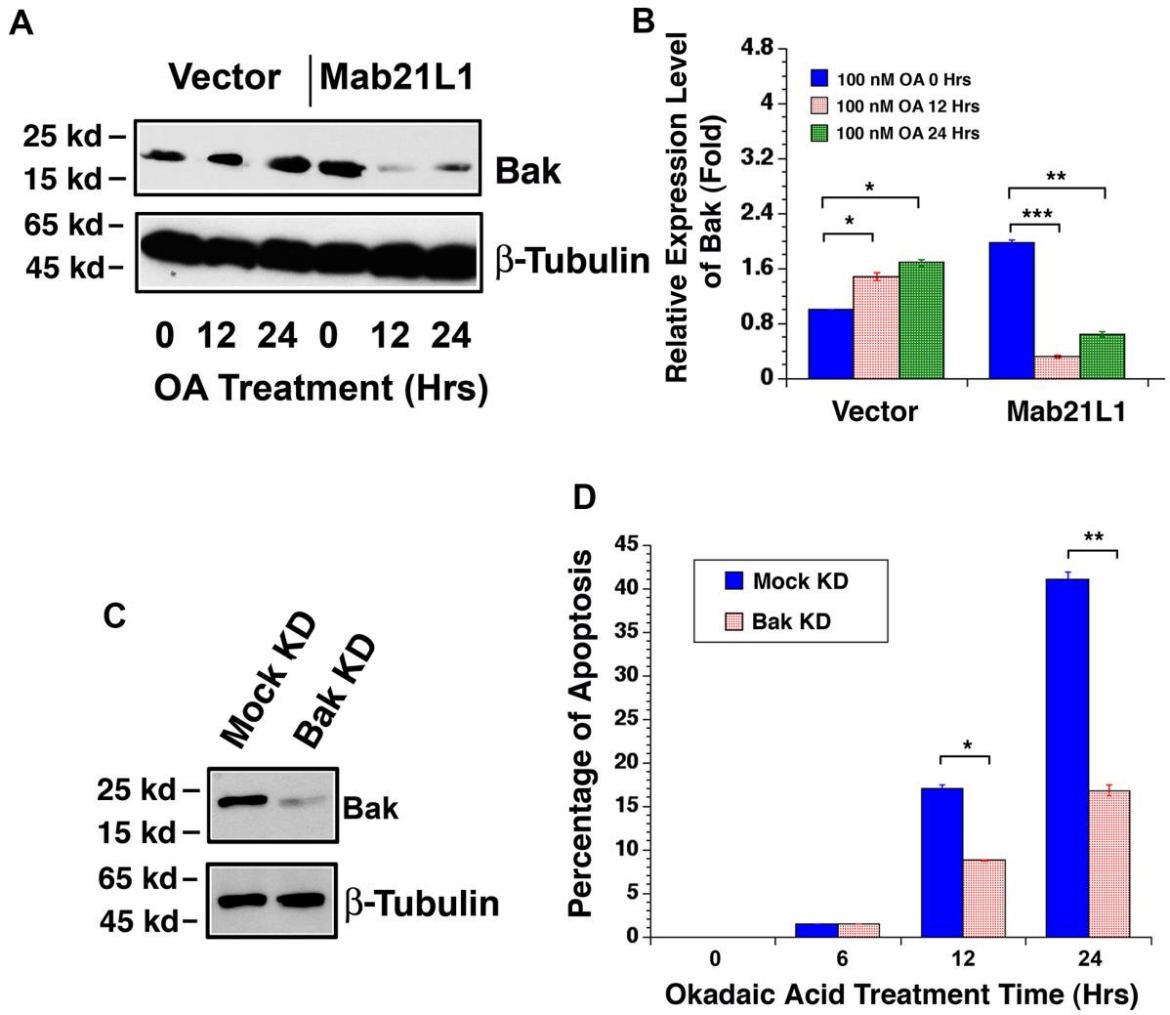
**Dynamic change of Bak levels in vector-αTN4-1 and MAB21L1-αTN4-1 cells in the absence and presence of 100 OA treatment and effect of Bak level on OA-induced apoptosis of αTN4-1 cells.** (**A**, **B**). Dynamic change of Bak levels in vector-αTN4-1 and MAB21L1-αTN4-1 cells in the absence and presence of 100 OA treatment. Both vector-αTN4-1 and MAB21L1-αTN4-1 cells were grown to about 90% confluence and then subjected to 100 nM OA treatment for 12 and 24 hrs. Thereafter, the cells were harvested for extraction of total proteins which were used for analysis of Bak expression by Western blot analysis (**A**). Quantitative results of the Bak protein expression levels were analyzed by Image J software (**B**). Note that in the MAB21L1-αTN4-1 cells, the Bak protein expression level was much higher than that in the vector-αTN4-1 cell clone in the absence of 100 nM OA treatment. During OA treatment for 12 and 24 hrs, however, Bak protein was upregulated in vector-αTN4-1 cells. In contrast, in MAB21L1-αTN4-1 cells, Bak protein was significantly degraded. N=3. *p<0.05, ** *p*<0.01, *** *p*<0.001. (**C**, **D**) Effect of Bak level on OA-induced apoptosis of αTN4-1 cells. Vector-αTN4-1 cells were used as Bak knockdown with CRSPR/Cas9 technology (see Materials and Methods). Both mock and Bak KD clones were verified with Western blot analysis (**C**). The two types of cells were then subjected to 100 nM OA treatment for 12 and 24 hrs, and the apoptosis rate was determined with live/dead assays (**D**). N=3. *p<0.05, ** *p*<0.01.

Since Mab21L1 may prevent p53 hyperphosphorylation through upregulation of αB-crystallin, we next tested if Mab21L1 have any effect on the downstream targets of p53 during okadaic acid-induced apoptosis. As shown in Figure 7A, 7B, in Mab21L1 expression cells, Bak expression was relatively higher than that in vector transfected cells. However, during okadaic acid treatment, we observed that Bak was upregulated in vector-transfected cells. However, in Mab21L1 expression cells, okadaic acid-induced upregulation of Bak was abrogated. Next, we silenced Bak expression in mouse lens epithelial cells, αTN4-1 using CRISPR/Cas9 technology [49] (Figure 7C). As shown in Figure 7D, silence of Bak expression significantly attenuated okadaic acid-induced apoptosis. Thus, Mab21L1 can downregulate Bak level to suppress okadaic acid-induced apoptosis.

### Mab21L1 promotes Mcl-1 expression during OA-induced apoptosis and overexpression of Mcl-1 suppresses OA-induced apoptosis

**Figure 8:**
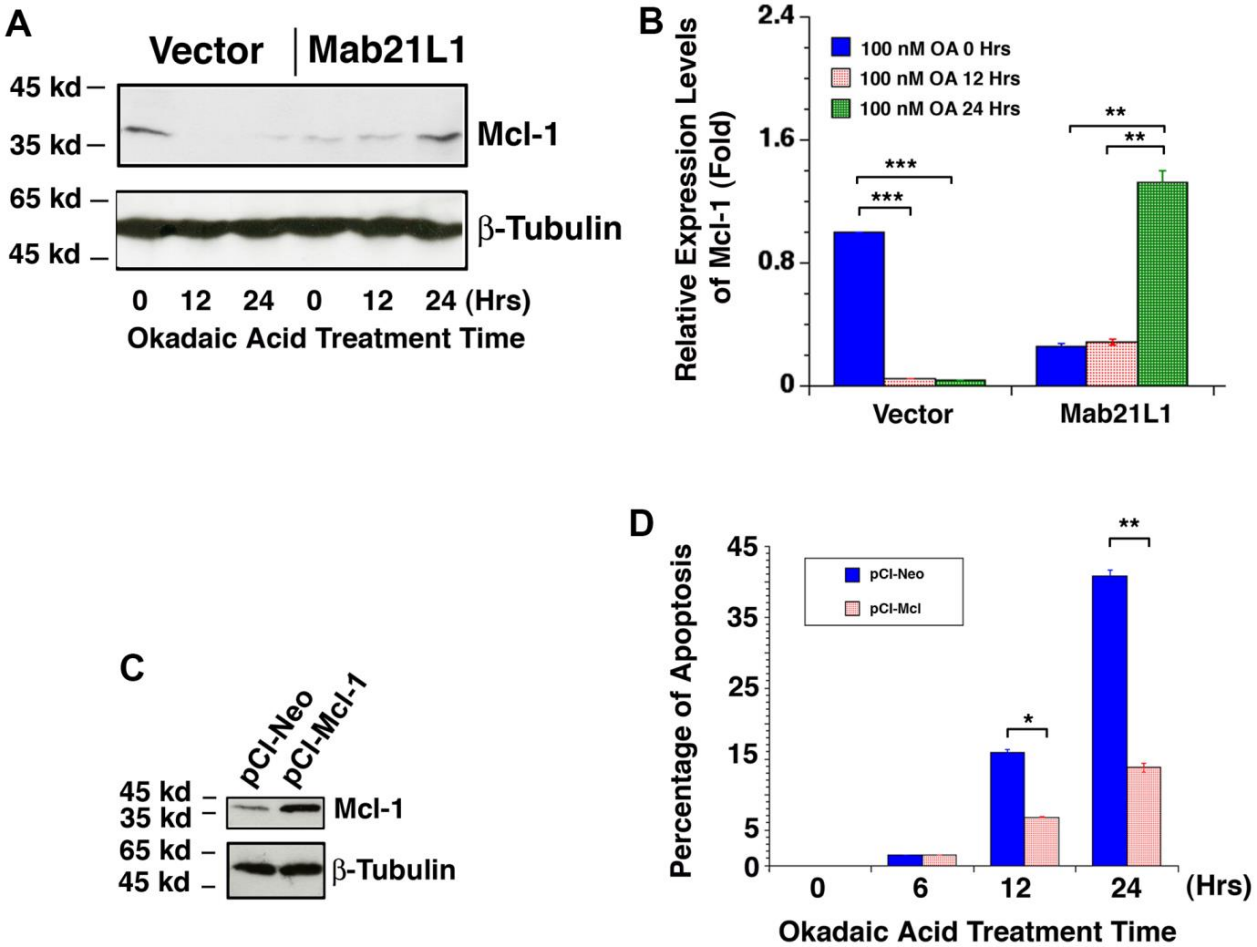
**Dynamic change of Mcl-1 levels in vector-αTN4-1 and MAB21L1-αTN4-1 cells in the absence and presence of 100 OA treatment and effect of Mcl-1 level on OA-induced apoptosis of αTN4-1 cells.** (**A**, **B**) Dynamic change of Mcl-1 levels in vector-αTN4-1 and MAB21L1-αTN4-1 cells in the absence and presence of 100 OA treatment. Both vector-αTN4-1 and MAB21L1-αTN4-1 cells were grown to about 90% confluence and then subjected to 100 nM OA treatment for 12 and 24 hrs. Thereafter, the cells were harvested for extraction of total proteins which were used for analysis of Mcl-1 expression by Western blot analysis (**A**). Quantitative results of the Mcl-1 protein expression levels were analyzed by Image J software (**B**). Note that in the MAB21L1-αTN4-1 cells, the Mcl-1 protein expression level was much lower than that in the vector-αTN4-1 cell clone in the absence of 100 nM OA treatment. During OA treatment for 12 and 24 hrs, however, Mcl-1 protein was down-regulated to background level in vector-αTN4-1 cells. In contrast, in MAB21L1-αTN4-1 cells, Mcl-1 protein was significantly upregulated after 24 h treatment by OA. N=3. ** *p*<0.01, *** *p*<0.001. (**C**, **D**) Effect of Mcl-1 level on OA-induced apoptosis of αTN4-1 cells. Both pCI-αTN4-1 and pCI-Mcl-1-αTN4-1 cell clones were verified with Western blot analysis (**C**). The two types of pCI-αTN4-1 and pCI-Mcl-1-αTN4-1 cells were then subjected to 100 nM OA treatment for 12 and 24 hrs, and the apoptosis rate was determined with live/dead assays (**D**). N=3. *p<0.05, ** *p*<0.01.

To further understand how Mab21L1 regulates survival of lens epithelial cells, we examined the dynamic change of another Bcl-2 family member, the *mcl-1* gene encoding an important anti-apoptotic regulator [60–63]. A previous study reported that Mcl-1 expression is related to p53 activity in a reverse relationship [99]. As shown in Figure 8A, 8B, in Mab21L1 expression cells, Mcl-1 expression was slightly lower than that in vector transfected cells. However, during okadaic acid treatment, we observed that Mcl-1 was downregulated in vector-transfected cells. However, in Mab21L1 expression cells, okadaic acid induced upregulation of Mcl-1. Next, we overexpressed Mcl-1 in mouse lens epithelial cells αTN4-1 using the vector, pCI-Mcl-1 (Figure 8C). As shown in Figure 8D, overexpression of Mcl-1 significantly suppressed OA-induced apoptosis. Thus, Mab21L1 can upregulate Mcl-1 level to attenuate OA-induced apoptosis.

## DISCUSSION

**Figure 9:**
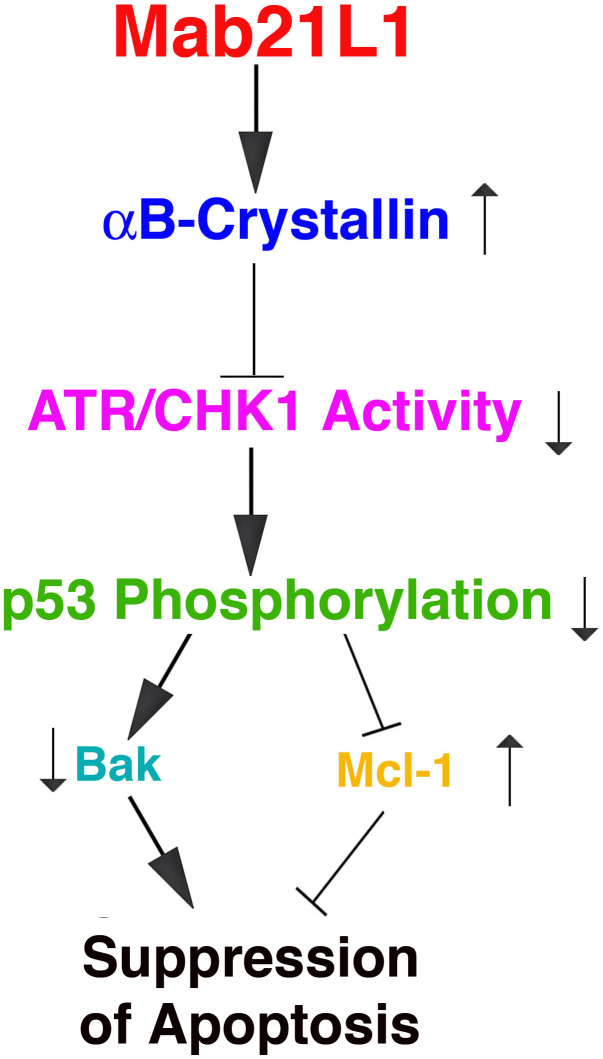
**Diagram to show the mechanism mediating Mab21L1 promotion of survival of lens epithelial cells.** Mab21L1 promotes survival of lens epithelial cells through upregulation of αB-crystallin to suppress the ATR/CHK1/p53 pathway and thus downregulate Bak expression but enhance Mcl-1 expression. As a result, MAB21L1 can suppress stress (okadaic acid or UVA)-induced apoptosis.

In the present study, we have demonstrated the followings: 1) Overexpression of human *mab21L1* gene cDNA in mouse lens epithelial cells, αTN4-1 significantly promotes survival of lens epithelial cells under treatment by okadaic acid; 2) Overexpression of human *mab21L1* gene cDNA in αTN4-1 cells upregulates expression of αB-crystallin gene; 3) αB-crystallin prevents UVA-induced hyperphosphorylation of the tumor suppressor p53 at S-20 and S-37; 4) αB-crystallin inhibits UVA-induced activation of the check kinase 1 (CHK1) and its upstream kinase ATR; 5) Overexpression of human *mab21L1* gene cDNA in αTN4-1 cells downregulates the pro-apoptotic factor Bak but upregulates expression of the pro-survival regulator Mcl−1 during okadaic acid treatment. Together, our results demonstrate that MAB21L1 promotes survival of lens epithelial cells through upregulation of αB-crystallin to suppress ATR/CHK1/p53 pathway. (Figure 9).

### Mab21L1 positively regulates αB-crystallin to promote survival of lens epithelial cells

The *mab21* gene was first identified as a cell fate determination gene that regulates sensory ray morphogenesis in male *C. elegans* [1]. Later it was found that Mab21L1 is a lens lineage-specific transcription factor [100]. It has an important role in regulating lens development [5, 7–9, 11]. Mutations in human Mab21L1 gene causes aberrations in lens ectoderm morphogenesis and lead to congenital cerebellar, ocular, craniofacial and genital (COFG) syndrome [101, 102]. The ocular abnormalities include microphthalmia, coloboma and cataracts [7, 9]. Similar to human *mab21L1* gene mutations, Mab21L1-deficient mice display severe cell-autonomous defects in lens placode invagination due to impaired cell proliferation and survival [5] and other deficiency [6, 101]. Using mouse lens epithelial cells, αTN4-1 and the stable lines expressing P3X-Flag-CMV-10 vector, or human *mab21L1* gene cDNA, here we demonstrated that human *mab21L1* gene cDNA-expression lens epithelial cells are much resistant against stress condition of okadaic acid treatment than the vector-transfected cells (Figure 2). Mechanistically, we demonstrated that Mab21L1 is capable of upregulating αB-crystallin, a strong anti-apoptotic regulator (See below discussion for more details).

How might Mab21L1 up-regulates αB-crystallin? In a recent study using single cell sequencing strategy, Yamada et al. (2021) [8] demonstrated that in Mab21L1 knockout mice, 61 genes were upregulated and 131 genes were downregulated in comparison with the wild type mice. Among these down-regulated genes are two transcription factors: Maf and Sox2 [8]. Since previous studies have shown that both Maf and Sox2 can positively regulates expression of αB-crystallin [100, 103, 104], it is plausible that Mab21L1 may upregulate αB-crystallin through Maf and Sox-2. Alternatively, Mab21L1 may directly bind to the promoter of αB-crystallin gene to control its expression. Whether this is the case is currently under investigation.

### αB-crystallin represses ATR/CHK1/p53 pathway to inhibit apoptosis

As an anti-apoptotic regulator, αB-crystallin can prevent cells from induced-apoptosis in several mechanisms based on the studies from us and several other laboratories [38, 51, 54, 82–89]. First, it can suppress the maturation of the members of the caspase family [38, 51, 83–84]. Second, it can also interact with members of the Bcl-2 family including Bax and Bcl-Xs to prevent their translocation into mitochondria [54]. Third, αB-crystallin was found to negatively regulate the Ras/Raf/MEK/ERK signaling pathway to prevent UVA-induced apoptosis [40, 82].

Previous studies have shown that αB-crystallin can also bind to the tumor suppressor, p53 [105]. However, how does αB-crystallin modulate p53 activity remains unknown. Here, using stable lines of human lens epithelial cells HLE expressing pEGFP-vector, or human αB-crystallin gene cDNA, we demonstrated that UVA induced hyperphosphorylation of p53 at S-20 and S-37 in pEGFP-HLE cells (Figure 4A–4D). This UVA-induced p53 hyperphosphorylation is significantly reduced in αB-crystallin expression cells, pEGFP-HαB-HLE (Figure 4A–4D). At present, seventeen phosphorylation sites have been identified [92]. Whether the remaining 15 phosphorylation sites besides S-20 and S-37 can be induced by UVA and whether αB-crystallin also has strong impact on the phosphorylation of the remaining phosphorylation sites remains to be investigated. Nevertheless, our study demonstrates that αB-crystallin can modulate p53 phosphorylation status at S-20 and S-37 to inhibit its activity.

It has been well established that p53 can be phosphorylated by check kinase 1 (CHK1) at S-20 and S-37 [93, 94]. Our results also showed that while in vector-transfected cells (pEGFP-HLE), UVA irradiation increased CHK1 activity, in αB-crystallin expression cells (pEGFP-HαB-HLE), however, both CHK1 protein level and activity were down-regulated under UVA irradiation (Figure 5). Since CHK1 is activated by ATR kinase [95–98], we also examined the differential activation of ATR in vector- and HαB-transfected cells. Our results demonstrated that while in vector-transfected cells, pEGFP-HLE, UVA induced upregulation of ATR activity from 20% to 50% under irradiation with 37.5KJ/m^2^ to 75 KJ/m^2^, respectively; in αB-crystallin expression pEGFP-HαB-HLE cells, in contrast, both ATR protein level and activity were slightly down-regulated under UVA irradiation of both doses (Figure 6). Together, out results demonstrate that αB-crystallin can repress ATR/CHK1/p53 pathway to inhibit p53-mediated apoptosis.

### αB-crystallin suppression of ATR/CHK1/p53 contributes to Mab21L1 promotion of survival of lens epithelial cells

In the present study, we also observed that in Mab21L1 expression cells, expression of Bak is relatively higher than that in vector-transfected cells (Figure 7), and Mcl-1 expression, on the other hand, is relatively lower than that in vector-transfected cells (Figure 8). However, Mab21L1-induced upregulation of αB-crystallin can override the Bak upregulation and Mcl-1 downregulation to promote survival of lens epithelial cells in the absence of stress conditions.

Under okadaic acid treatment, however, we observed that Bak expression was upregulated and Mcl-1 expression was down-regulated in vector-transfected cells. In contrast, in Mab21L1 expression cells, Bak expression was significantly down-regulated and Mcl-1 was significantly upregulated (Figures 7A, 7B, 8A, 8B). Moreover, when Bak was knocked down or Mcl-1 was overexpressed, okadaic acid-induced apoptosis of αTN4-1 cells was significant inhibited (>50%, Figures 7C, 7D, 8C, 8D). This OA-induced Bak downregulation and Mcl-1 upregulation in Mab21L1 expression cells is closely related with its promotion of αB-crystallin expression. Under the same okadaic acid treatment condition, OA induced significant downregulation of αB-crystallin in vector-transfected cells (Figure 3). In Mab21L1 expression cells, however, OA-induced much less downregulation of αB-crystallin (Figure 3). Thus, Mab21L1-promoted upregulation of αB-crystallin through suppression of ATR/CHK1/p53 pathway, can promote survival of lens epithelial cells (Figure 9).

## MATERIALS AND METHODS

### Materials

Various molecular biology reagents were purchased from Invitrogen Life Technologies (Gaithersburg, MD, USA). All the oligos were purchased from Sangon Biotech Co., Ltd. (Shanghai, China). Protein size markers were purchased from GenStar Co., Ltd. (Beijing, China). Various antibodies were obtained from Cell Signaling Technology (Boston, MA, USA); Abcam Inc. (Cambridge, MA, USA); Santa Cruz Biotechnology, Inc. (Dallas, TX, USA); Sigma-Aldrich (St. Louis, MO, USA); Proteintech Co., Ltd. (Wuhan, China); Ray Biotech Co., Ltd. (Beijing, China). 

### Cell culture, plasmid construction and establishment of gene overexpression or knockout stable cell lines

Mouse lens epithelial cell line αTN4-1 and human lens epithelial cells (HLE) were cultured in Dulbecco’s modified Eagle’s medium (DMEM) supplemented with 10% fetal bovine serum (FBS; Atlanta Biologicals) and 1% penicillin/streptomycin in 5% CO_2_ at 37° C as described before [42, 49, 54, 59, 106, 107].

The CRISPR/Cas9-based gene KO vector pSp Cas9(BB)-2A-Puro (PX459) is a gift from Dr. Mengqing Xiang in Zhongshan Ophthalmic Center of Sun Yat-sen University. The sgRNA sequences used for Bak gene KO were: 5’-CAAGTTGTCCATCTCGGGGTTGG-3’ (target 1) and 5’ TCTTCACCAAGATCGCCTCCAGG 3’ (target 2) as described before [49]. The sgRNA was inserted into PX459 using the BbsI restriction sites. For expression vector, the construction of full-length cDNAs of Mab21L1 or Mcl-1 were cloned by PCR. The Mab21L1 cDNA was inserted into the expression vector, p3xFlag-CMV-10-Mab21L1, at the EcoRI and KpnI sites. The Mcl-1 cDNA was subcloned into the pCI-Neo Vector, at the EcoR1 and Sal I sites.

For establishment of stable cell lines, αTN4-1 cells were transfected with the above plasmids using Lipofectamine 3000 (Life Technologies) according to the manufacturer’s instructions. Forty-eight hours after transfection, cells stably expressing the plasmids were selected by 1.0 μg/mL puromycin for PX459, and 600 μg/ml G418 for p3×FLAG-CMV-10 and pCI-Neo. About 4 weeks, individual clones for the stable cell lines were established and confirmed by western blot analysis and DNA sequencing [49, 106, 107].

pEGFP-HLE and pEGFP-HαB-HLE were established as described before [54, 106]. These cells were cultured in DMEM supplemented with 10% fetal bovine serum (FBS; Atlanta Biologicals), 600 μg/ml G418 and 1% penicillin/streptomycin in 5% CO_2_ at 37° C.

### UVA irradiation

The UVA irradiation was conducted with similar facility described previously [82], which produces an energy level of approximately 1 mW cm−2 S−1 with the wavelength ranging from 320 to 400 nm. The amount of UVA light reaching the lens is between 0.1 and 1 mW cm−2 [108]. The total energy received by the lens epithelial cells was 37.5 kJ m−2 (3.75 J cm−2), or 75 kJ m−2 (7.5 J cm−2). Briefly, the irradiation was conducted with an uncovered 100 mm culture dish containing 100% confluence HLE cell with 10 ml DMEM plus 10% FBS. We conducted UVA irradiation in the presence of culture medium to avoid the cellular responses to nutritional shock (for example, withdrawal of growth factors in serum). Under the present irradiation condition, UVA may elicit production of hydrogen peroxide according to a previous study [108]. A dose of 75 kJ m−2 corresponds to the amount of UVA which our naked skin receives in approximately 1.5 hours of exposure to sunshine in a typical summer day (90° C) in August at noon time.

### Protein extraction and Western blot analysis

Total proteins were extracted by RIPA buffer (1% NP-40, 1% sodium deoxycholate, 0.1% SDS, 50 mM Tris-HCl PH8.0, 150 mM NaCl) supplemented with the Protease Inhibitor Cocktail, then cell lysates were sonicated and centrifuged at 13000×rpm for 10 min at 4° C as described [106, 107]. The supernatants were transferred to new tubes. Fifty micrograms of total proteins in each sample was separated by 10% or 12 % SDS-polyacrylamide gel and transferred to PVDF membranes. The protein blots were blocked with 5% nonfat milk in TBST (10 mM Tris HCl, pH8.0, 150 mM NaCl, 0.05% Tween-20) and further incubated with primary antibodies overnight at 4° C. Primary antibodies used in western blot were shown in Supplementary Table 1. The horseradish peroxidase-conjugated secondary antibodies (CST; 7077 and 7074) were then applied for one hour at room temperature. Immunoreactivity was detected with a chemilluminescence detection kit (ECL Ultra; New Cell and Molecular Biotech Co., Ltd.) and the blots were visualized using a Tanon chemiluminescence system (China).

### Apoptosis assays

Cell apoptosis was determined by Hoechst staining [38, 51] and Live/Dead Viability/Cytotoxicity [49]. Cells were seeded into 60 mm petri dish (for Hoechst staining) or into 6-well plates about 90% confluence, and then treated with 100 nM Okadaic acid for different time as indicated in the figures to induce cell apoptosis. The apoptotic nature of the treated cells was further verified by Hoechst staining as previously described [51]. The Live/Dead Viability/Cytotoxicity Kit (L3224; Thermo Fisher) was used to distinguish live and dead cells [49]. Live cells are characterized by the presence of ubiquitous intracellular esterase activity, and revealed by calcein AM. The polyanionic dye calcein is well retained within live cells, producing an intense uniform green fluorescence in live cells. EthD-1 enters cells with damaged membranes and displays red fluorescence upon binding to nucleic acids, thereby producing a bright red color in dead cells and EthD-1 is excluded by the membrane of live cells. The images were captured with a Zeiss microscope.

### Statistics

All results shown are reported as the mean ± standard deviation (SD). Significance was calculated using the unpaired two-tailed t test. Differences were considered statistically significant at P < 0.05.

### Data availability statement

All data are available upon reasonable request.

## Supplementary Material

Supplementary Figure 1

Supplementary Table 1
