# Supplementary material for: MAB21L1 promotes survival of lens epithelial cells through control of αB-crystallin and ATR/CHK1/p53 pathway
Source: Aging (Albany NY). 2022 Aug 10;14(15):6128–48. doi: 10.18632/aging.204203 (PMC9417230; doi:10.18632/aging.204203)
Supplement: Supplementary Figure 1 [file aging-14-204203-s001.pdf]

SUPPLEMENTARY FIGURE

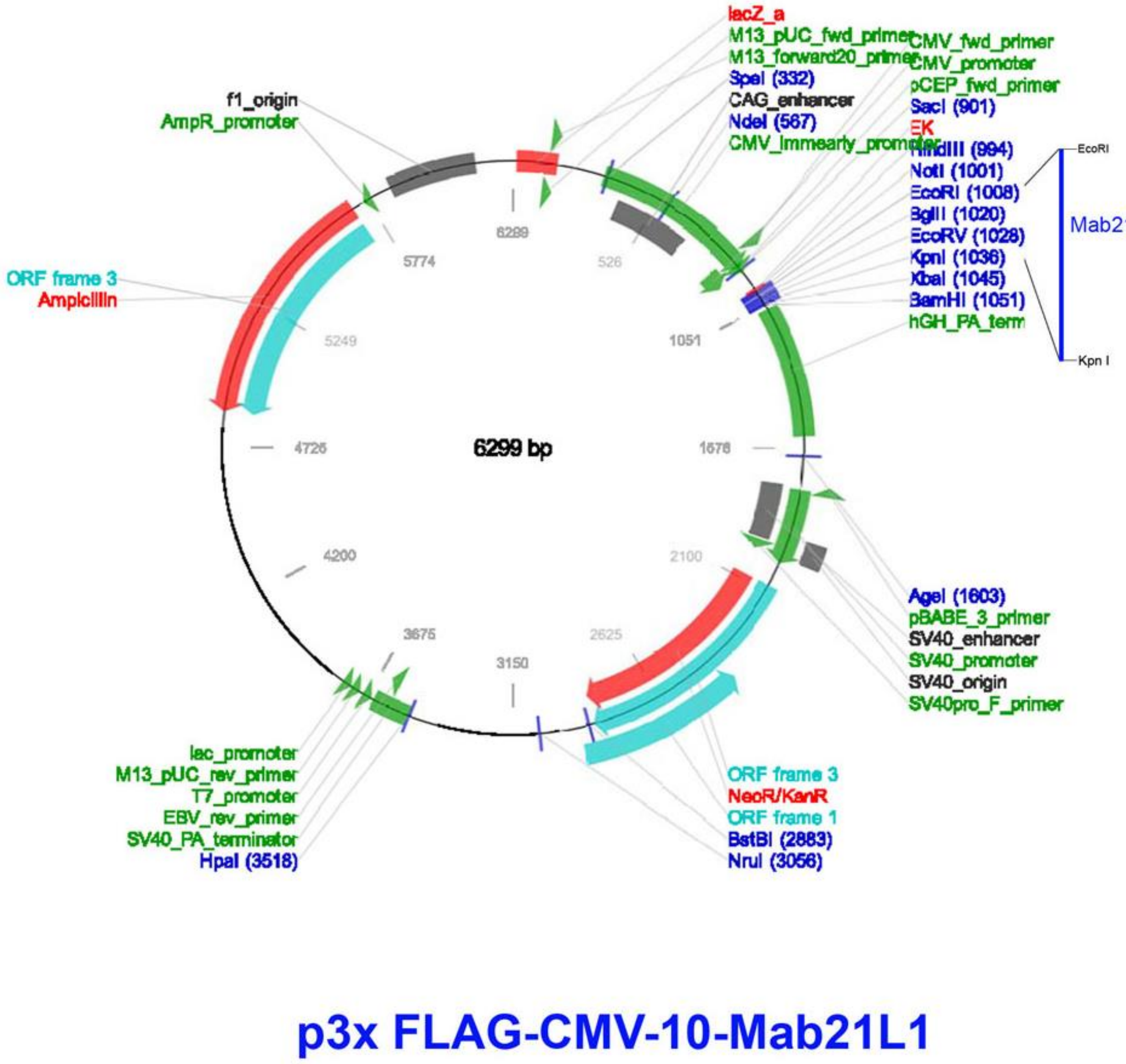

Supplementary Figure 1. Diagram to show the P3X-Flag-CMV-10-MAB21L1 plasmid. The Human coding sequence of MAB21L1 was cloned and inserted into the EcoR1 and Kpn1 restriction enzyme sites of the P3X-Flag-CMV-10 vector.
