# Supplementary material for: MAB21L1 promotes survival of lens epithelial cells through control of αB-crystallin and ATR/CHK1/p53 pathway
Source: Aging (Albany NY). 2022 Aug 10;14(15):6128–48. doi: 10.18632/aging.204203 (PMC9417230; doi:10.18632/aging.204203)
Supplement: Supplementary Table 1 [file aging-14-204203-s002.pdf]

SUPPLEMENTARY TABLE

Supplementary Table 1. Chemicals and antibodies used in the present study.

Chemicals and antibodies	Vendors and categories
Okadaic Acid	LC Laboratory, O5857
Anti-Flag	Sigma-Aldrich, F1804
Anti-Mab21L1	Abcam, ab154311
Anti-GAPDH	Proteintec, 60004
Anti-β-Tubulin	Proteintec, 66240
Anti-β-Actin	Proteintec, 66009
Anti-αB	Gift of Dr. Joseph Horwitz from UCLA
Anti-T-p53	CST, 9282/2524
Anti-p-p53-S20	CST, 9287
Anti-p-p53-S37	CST, 9289
Anti-T-CHK1	CST, 2360
Anti-p-CHK1-S345	CST, 2348
Anti-T-ATR	CST, 2790
Anti-p-ATR-S428	CST, 2853
Anti-Bak	CST, 3814
Anti-Mcl-1	CST, 4572
